# Preparation of PPy-Coated MnO_2_ Hybrid Micromaterials and Their Improved Cyclic Performance as Anode for Lithium-Ion Batteries

**DOI:** 10.1186/s11671-017-2286-3

**Published:** 2017-09-02

**Authors:** Lili Feng, Yinyin Zhang, Rui Wang, Yanli Zhang, Wei Bai, Siping Ji, Zhewen Xuan, Jianhua Yang, Ziguang Zheng, Hongjin Guan

**Affiliations:** 1 School of Chemistry and Environment, Yunnan Minzu University, Kunming, 650500 China; 2 Key Laboratory of Resource Clean Conversion in Ethnic Regions, Education Department of Yunnan, Yunnan Minzu University, Kunming, 650500 China

**Keywords:** Manganese dioxide, PPy, Lithium-ion battery, Anode material

## Abstract

MnO_2_@PPy core-shell micromaterials are prepared by chemical polymerization of pyrrole on the MnO_2_ surface. The polypyrrole (PPy) is formed as a homogeneous organic shell on the MnO_2_ surface. The thickness of PPy shell can be adjusted by the usage of pyrrole. The analysis of SEM, FT-IR, X-ray photoelectron spectroscopy (XPS), thermo-gravimetric analysis (TGA), and XRD are used to confirm the formation of PPy shell. Galvanostatic cell cycling and electrochemical impedance spectroscopy (EIS) are used to evaluate the electrochemical performance as anode for lithium-ion batteries. The results show that after formation of MnO_2_@PPy core-shell micromaterials, the cyclic performance as anode for lithium-ion batteries is improved. Fifty microliters of PPy-coated caddice-clew-like MnO_2_ has the best cyclic performances as has 620 mAh g^−1^ discharge specific capacities after 300 cycles. As a comparison, the discharge specific capacity of bare MnO_2_ materials falls to below 200 mAh g^−1^ after 10 cycles. The improved lithium-storage cyclic stability of the MnO_2_@PPy samples attributes to the core-shell hybrid structure which can buffer the structural expansion and contraction of MnO_2_ caused by the repeated embedding and disengagement of Li ions and can prevent the pulverization of MnO_2_. This experiment provides an effective way to mitigate the problem of capacity fading of the transition metal oxide materials as anode materials for (lithium-ion batteries) LIBs.

**Electronic supplementary material:**

The online version of this article (10.1186/s11671-017-2286-3) contains supplementary material, which is available to authorized users.

## Background

Since 3d transition metal oxides (MO; where M is Fe, Co, Ni, and Cu) were proposed to serve as high theoretic capacity anodes for lithium-ion batteries by Tarascon et al. [1], many efforts have been made in preparing micro/nano-metal oxides with various morphologies and researching their electrochemical performance as anode for lithium-ion batteries [2–6]. For examples, Zhu’s research group had made monodisperse Fe_3_O_4_ and γ-Fe_2_O_3_ microspheres via a surfactant-free solvothermal method [3]. They had a high initial discharge capacity of 1307 and 1453 mAh g^−1^, respectively. After 110 cycles, the discharge capacity remained at 450 mAh g^−1^ for Fe_3_O_4_ and 697 mAh g^−1^ for γ-Fe_2_O_3_. Hongjing Wu et al. had prepared uniform multi-shelled especially quintuple-shelled NiO hollow spheres by a simple shell-by-shell self-assembly hydrothermal treatment. The merit of this research made a significant contribution to the synthetic methodology of multi-shelled hollow structures. But the lithium storage performances of the NiO hollow spheres were not very excellent [4]. MnO_2_ possess high theoretically gravimetric lithium storage capacity of about 1230 mAh g^−1^; therefore, many researches are made to the design, synthesis, and applications of MnO_2_ anodes for lithium-ion battery [7–10]. For instance, Chen’s research group had made γ-MnO_2_ with hollow microspherical shape and nanocubic shape [11]. After 20 cycles, the discharge capacities of the nanocubes and microspheres were 656.5 and 602.1 mAh g^−1^. In addition, they had made many researches on MnO_2_ materials for lithium-ion battery from the year 2000 to now [12, 13]. We also studied the applications of MnO_2_ anodes for lithium-ion battery, but the discharge specific capacity of bare MnO_2_ materials felled so fast to below 200 mAh g^−1^ after 10 cycles [14].

Although transition metal oxides materials have large theoretical specific capacities, all these materials including MnO_2_ anodes are generally plagued by rapid capacity fading. The reasons for the poor cycling stability are as follows: (1) the electronic conductivity of transition metal oxides materials are usually low, and the electron or ion have difficulties in the diffusion process, resulting in irreversible electrode reaction and fast capacity decay. (2) After charge/discharge cycles, transition metal oxides suffer from enormous mechanical stress and pulverize, leading to electrical contact loss between active particles and current collector. The transition metal oxide particles without electrical contact can no longer participate in the charge/discharge cycles, resulting in capacity fading [15, 16].

Shell coating is an effective strategy to improve the cycling stability. In this structure, to a certain extent, the shell can buffer the structural expansion and contraction of metal oxide materials caused by the repeated embedding and disengagement of the Li ions. For the moment, carbon coating, organic conducting polymer coating, graphene hybrid, and other inorganic compound coating have been used [17, 18]. For instance, Yin et al. prepared polypyrrole (PPy)-coated CuO nanocomposites. The core-shell sample had a high reversible capacity of 760 mAh g^−1^ which was much better than those of the bare CuO sample [19]. Li et al. prepared graphene-wrapped MnO_2_ nanoribbons. The reversible specific discharge capacity reached 890 mAh g^−1^ at 0.1 A g^−1^ after 180 cycles. Therefore, it is necessary and urgent to make PPy shell coating on MnO_2_ materials to improve the cyclic stability as anode for lithium-ion batteries [20].

In the present work, to improve the cyclic performance of MnO_2_ materials as anode for lithium-ion batteries, polypyrrole (an organic conducting polymer) coating had been prepared by chemical polymerization. As a result, the cyclic performance was improved after formation of MnO_2_@PPy core-shell micromaterials. This experiment provides an effective way to mitigate the problem of capacity fading of the transition metal oxides materials as anode materials for (lithium-ion batteries) LIBs.

## Methods

### Preparation of Samples

All reagents were of analytical grade and purchased from the Shanghai Chemical Company. The pyrrole was purified by decompressional distillation prior to use and stored at 0–5 °C and guarded against exposure to light to prevent residual polymerization. Other reagents were used without further purification.

The MnO_2_ micromaterials were prepared using the similar method described by Yu et al. [14, 21] as some modification. To prepare caddice-clew-like MnO_2_ micromaterial, 1.70 g MnSO_4_·H_2_O was dissolved in 15 mL distilled water with vigorous stirring. When the solution was clear, 20 mL aqueous solution containing 2.72 g K_2_S_2_O_8_ were added to the above solution under continuous stirring. Then, the resulting transparent solution was transferred into a Teflon-lined stainless steel autoclave (50 mL) of 80% capacity of the total volume. The autoclave was sealed and maintained at 110 °C for 6 h. After the reaction was completed, the autoclave was allowed to cool to room temperature naturally. The solid black precipitate was filtered, washed several times with distilled water to remove impurities, and then dried at 80 °C in air for 3 h. The obtained caddice-clew-like MnO_2_ micromaterial was collected for the fabrication of PPy-coated MnO_2_ materials. Urchin-like MnO_2_ micromaterial was prepared by the similar method; after adding 1.70 g MnSO_4_·H_2_O and 2.72 g K_2_S_2_O_8_ into 35 mL distilled water, 2 mL H_2_SO_4_ was then added.

The MnO_2_@PPy hybrid micromaterials were prepared by chemical polymerization of pyrrole on the MnO_2_ surface using sodium benzenesulfonate (BSNa) as surfactant and FeCl_3_ as oxidant. The molar ratio of monomer pyrrole to BSNa was 3:1. First, 0.2 g MnO_2_ was dispersed into a beaker containing 50 mL of 0.01 mol L^−1^ BSNa aqueous solution and stirred for 0.5 h. The mixture was put in an ice/water bath (0–5 °C) under stirring. Then, a certain amount of pyrrole was added to the mixture. After stirring for 0.5 h, a small amount of FeCl_3_ solution was dropwise added into the aqueous solution to start the polymerization process. The gradual change of color from light black to deep black indicated the formation of PPy. The mixture was kept at 0–5 °C under stirring for 12 h to form MnO_2_@PPy core-shell micromaterials. The thickness of PPy was controlled by pyrrole usage. Finally, the obtained composite was filtered, washed with water and ethanol, and then dried under vacuum at 60 °C for 4 h.

### Characterization of Samples

The morphological investigations of SEM images and energy dispersive spectroscopy (EDS) were taken on a scanning electron microscope (QUANTA-200 America FEI Company). The crystallographic structures of the products were determined with XRD which were recorded on a Rigaku D/max-2200/PC with Cu target at a scanning rate of 7°/min with 2θ ranging from 10° to 70°. Fourier transform infrared (FT-IR) spectra of the MnO_2_@PPy hybrid micromaterials palletized with KBr were performed on a Nicolet IS10 spectrometer. Thermo-gravimetric analysis (TGA) was also used to determine the weight loss of MnO_2_@PPy hybrid micromaterials at 10 °C/min from 25 to 800 °C in air (MELER/1600H Thermogravimetric Analyzer). X-ray photoelectron spectroscopy (XPS) measurements were recorded on a Ulvac-PHI, PHI5000 Versaprobe-II X-ray photoelectron spectroscope, using Al Kα X-rays as the excitation source. The binding energy obtained in the XPS analysis was calibrated against the C1s peak at 284.8 eV.

### Cell Assemply and Electrochemical Studies

Electrochemical lithium-storage properties of the synthesized products were measured by using CR2025 coin-type test cells assembled in a dry argon-filled glove box. To fabricate the working electrode, a slurry consisting of 60 wt.% active materials, 10 wt.% acetylene black, and 30 wt.% poly-vinylidene fluoride (PVDF) dissolved in *N*-methyl pyrrolidinone was casted on a copper foil, dried at 80 °C under vacuum for 5 h. A lithium sheet was served as counter and reference electrode, while a Celgard 2320 membrane was employed as a separator. The electrolyte was a solution of 1 M LiPF_6_ in ethylene carbonate (EC)-1,2-dimethyl carbonate (DMC) (1:1 in volume). Galvanostatical charge-discharge experiments were performed by Land electric test system CT2001A (Wuhan Land Electronics Co., Ltd.) at a current density of 0.2 C between 0.01 and 3.00 V (versus Li/Li^+^). When calculating the specific capacity of MnO_2_@PPy core-shell micromaterials, the mass of PPy was included. Electrochemical impedance spectroscopy (EIS) measurements were performed on an electrochemical workstation (CHI604D, Chenhua, Shanghai), and the frequency ranged from 0.1 Hz to 100 KHz with an applied AC signal amplitude of 5 mV.

## Results and Discussion

### Morphological Features of Samples

**Fig. 1:**
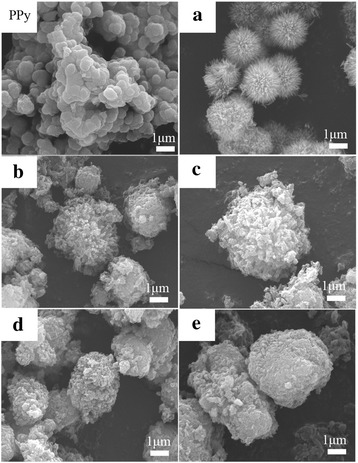
SEM images of PPy coated urchin-like MnO_2_ sample. In the top left-hand corner is pure PPy, **a** urchin-like MnO_2_ sample, **b** 10 μL, **c** 20 μL, **d** 30 μL, and **e** 50 μL pyrrole-coated urchin-like MnO_2_ sample. The scale bar is 1 μm

**Scheme 1:**
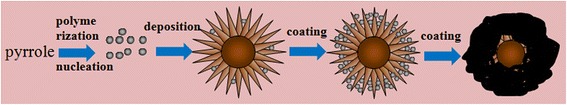
Schematic illustration of the formation mechanism proposed for MnO_2_@PPy material

The morphologies of pure PPy sample, sea-urchin-like MnO_2_ sample, and the MnO_2_@PPy hybrid micromaterials with different pyrrole polymerization amount are characterized by SEM measurements. As shown in Fig. 1, the pure PPy sample has a sphere shape with about 800 nm in diameter and tends to agglomerate together as layered rocks. The urchin-like MnO_2_ sample is shown in Fig. 1a. The MnO_2_ micromaterial is a uniform sea-urchin-like shape with a diameter of approximately 3 μm, which consists of several straight and radially grown nanorods with uniform length of about 1 μm. The evolution of the morphologies of MnO_2_@PPy hybrid micromaterials is shown in Fig.1b–e. When the amount of pyrrole is small, the PPy first nucleates and then embeds into the gap of needle-like nanorods of MnO_2_ samples. The needle-like nanorods in Fig. 1b are obviously wider than those showed in Fig. 1a. When the amount of pyrrole increases to 20 μL, the nanorod structure still exists but not obvious. As pyrrole quantity increases to 30 μL, the needle-like nanorod structure of MnO_2_ micromaterials disappears completely and become spherical in shape. When pyrrole quantity further increases (Fig. 1e), the PPy shell becomes very thick. Scheme 1 illustrates the possible formation processes for the MnO_2_@PPy hybrid micromaterials. In the first stage, a tiny crystal nucleus of PPy generates from monomer pyrrole by the oxidation of FeCl_3_. Then, the crystal nucleus deposits into the gap between the thorns on the surface of the “urchin.” With the continuous polymerization of PPy, the gap between the thorns is gradually filled up. At the end, the whole “urchin” is uniformly coated by PPy. The low magnification SEM images of MnO_2_@PPy hybrid micromaterials in Additional file 1 confirm that PPy shell is formed uniformly on MnO_2_@PPy sample.

In this work, caddice-clew-like MnO_2_ micromaterial is also coated by PPy using the similar method. The SEM morphologies are shown in Additional file 1: Supporting information 1. The caddice-clew-like MnO_2_ micromaterial is nanowire shaped and aggregates into 2–4 μm diameter spheres which look like a caddice-clew. When the amount of pyrrole is small, the PPy first forms as small particles and adheres on the surface of the MnO_2_ samples. With the amount of pyrrole increasing, PPy gradually cover the caddice-clew-like MnO_2_ completely to form a large block structure which looks like rocks.

The uniform coating of PPy is further verified by energy-dispersive X-ray (EDX) spectroscopy analysis (shown in Table 1). No carbon and nitrogen signals are detected on pure MnO_2_ sample. Significant amount of carbon and nitrogen signals are detected on PPy and MnO_2_@PPy samples due to the formation of the PPy shell. With increasing pyrrole usage, the content of carbon and nitrogen are also increased. The EDX data of caddice-clew-like MnO_2_@PPy samples are shown in Additional file 1: Supporting Information 4.

**Table 1:** EDX data for PPy, MnO_2_, and PPy-coated urchin-like MnO_2_ sample

Element	PPy At%	MnO_2_ At%	MnO_2_@PPy(10 μL) At%	MnO_2_@PPy(20 μL) At%	MnO_2_@PPy(30 μL) At%	MnO_2_@PPy(50 μL) At%
C	62.99	–	13.78	06.51	35.11	21.50
N	19.03	–	04.94	04.62	09.64	07.03
O	10.13	34.02	36.57	36.53	22.27	30.06
Mn	–	65.98	44.71	38.84	18.23	28.35

### FT−IR Analysis of Samples

**Fig. 2:**
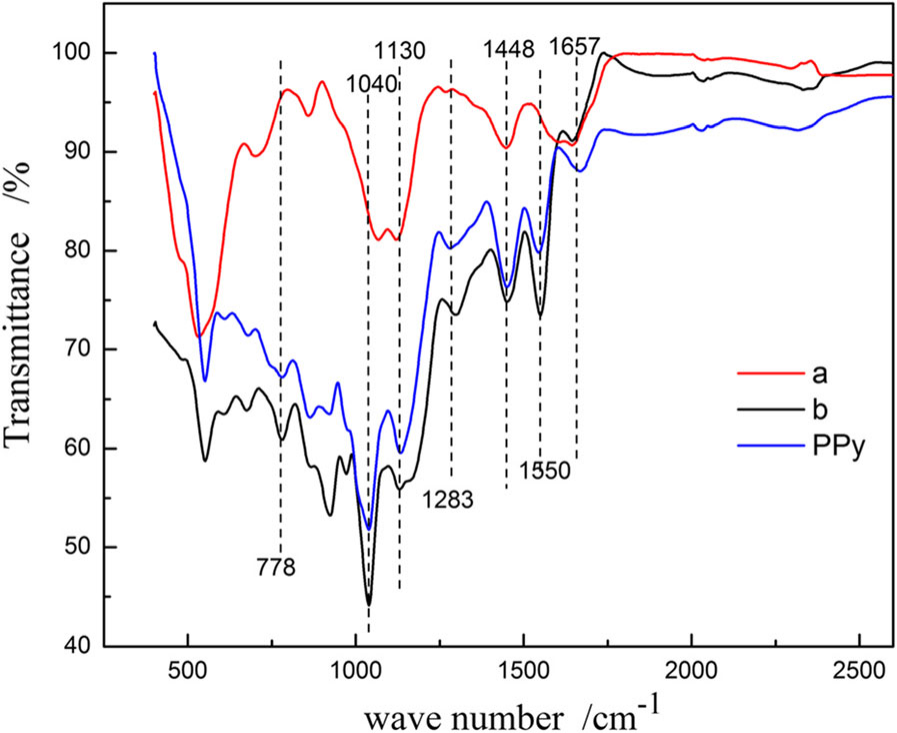
FT-IR spectra of (a) 30 μL PPy-coated urchin-like MnO_2_ sample and (b) 50 μL PPy-coated caddice-clew-like MnO_2_ sample and pure PPy

The structure features and compositions of the synthesized PPy and MnO_2_@PPy samples are further characterized by FT-IR spectroscopy (shown in Fig. 2). For all the MnO_2_@ PPy samples and PPy sample, the bands at 1550, 1448, 1283, and 1130 cm^−1^ are the characteristic peaks of the PPy rings. Among them, the peak at about 1550 cm^−1^ is due to C-C and C=C stretching, and the peak at about 1448 cm^−1^ is from C-N stretching of PPy. The peak at about 1130 cm^−1^ is due to the S=O stretching vibration peak that belongs to the BSNa, which indicates that the sulfonate ion is doped into the pyrrole ring. The ratio of I_1550_ and I_1448_ is usually ascribed to the conjugate and the doping degree of the PPy [22]. The higher the I_1550_/I_1448_ is the higher conjugate and doping degree of PPy is. That is, if I_1550_/I_1448_ is high, the conductivity of PPy should be better. The bands at 1550, 917, and 778 cm^−1^ of 30 μL PPy-coated urchin-like MnO_2_ sample are weaker than those of 50 μL PPy-coated caddice-clew-like MnO_2_ sample. So, the conductivity of PPy-coated caddice-clew-like MnO_2_ sample should be better, and the 50 μL PPy-coated caddice-clew-like MnO_2_ sample should have better lithium-storage performance. Bands at 1040 and 778 cm^−1^ are the in-plane and out-of-plane vibrations of C-H deformation of C_β_-H absorption band. No C_α_-H absorption band is observed in the spectrum, which indicates that the pyrrole ring is predominantly linked by α-α in PPy. The absorption band at 1657 cm^−1^ is due to the existence of water molecules in the products. Therefore, the FT-IR results prove that PPy shell is formed on MnO_2_@PPy sample.

### XPS Results

**Fig. 3:**
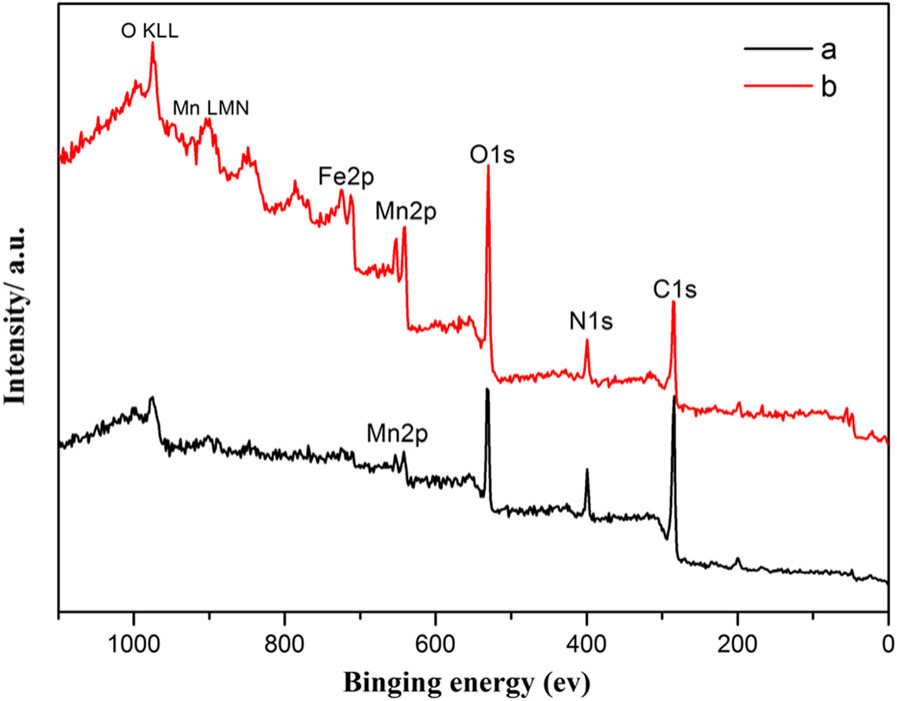
XPS spectra of (a) 30 μL PPy-coated urchin-like MnO_2_ sample and (b) 50 μL PPy-coated caddice-clew-like MnO_2_ sample

Usually, core-shell structure should be verified by TEM. However, the pure MnO_2_ sample here is too thick to take good TEM images. So, to verify the core-shell structure, we did XPS test and EDS test to verify the different components in the surface and the whole sample. For clarity, only the spectroscopy of 30 μL PPy-coated urchin-like MnO_2_ sample and 50 μL PPy-coated caddice-clew-like MnO_2_ sample are shown in Fig. 3. Others are in the Additional file 1: Supporting Information 5. The final results are listed in Table 2. The main binding energies (BE) of O1s, N1s, C1s, and Mn(2p1/2, 2p3/2) are determined to be 531.2, 398.9, 284.8 and 651.4 and 640.3 eV, respectively. The peaks at 973 and 901.6, and 848.9 eV are O KLL peaks (Auger peaks from oxygen atoms) and Mn LMM peaks (Auger peaks from Mn atoms). There is a few Fe or Cl detected by XPS, shown in Fig. 3. Here, the appearance of Fe or Cl signals is due to the use of FeCl_3_ as polymerization oxidant in preparing PPy shell. As can be seen in Table 2, the differences of EDS analysis and XPS analysis are distinct. In XPS analysis, the content of O, N, and C are much higher; the content of Mn is lower. The max analysis depth of XPS is about 5–10 nm. The strong O, N, and C peaks confirm that the MnO_2_ samples are covered by the PPy organic film (as described in the SEM paragraph).

**Table 2:** XPS data for urchin-like MnO_2_@PPy sample

Element	MnO_2_@PPy(10 μL) At%		MnO_2_@PPy(30 μL)At%		MnO_2_@PPy(50 μL)At%	
	EDX	XPS	EDX	XPS	EDX	XPS
C	07.39	49.1	35.11	65.8	21.50	69.4
N	–	1.30	09.64	11.0	07.03	12.8
O	16.24	46.8	22.27	22.0	30.06	16.4
Mn	13.61	2.90	18.23	0.50	28.35	1.10

### TGA Results

**Fig. 4:**
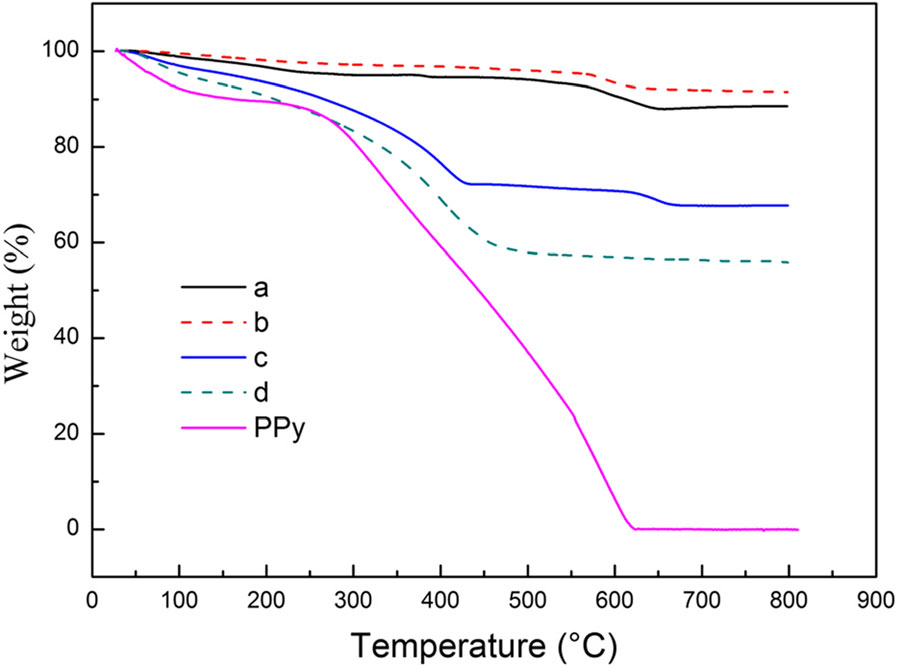
TGA curves of PPy and MnO_2_ samples. (**a**) urchin-like MnO_2_ sample, (**b**) caddice-clew-like MnO_2_ sample, (**c**) 30 μL PPy-coated urchin-like MnO_2_ sample, and (**d**) 50 μL PPy-coated caddice-clew-like MnO_2_ sample

To prove the PPy shell on the synthesized MnO_2_@PPy samples, TGA of bare MnO_2_ sample, bare PPy, and MnO_2_@PPy samples are carried out in air. Figure 4 is the TGA results. As can be seen from Fig. 4, the bare PPy powder displays two weight loss regions. The first weight loss about 12% in the temperature range of 60–260 °C can be attributed to the desorption of physisorbed water and removal of surface-absorbed solvents as mentioned in the previous literatures [19, 23, 24]. While the second weight loss about 88% in the range of 260–600 °C is ascribed to the oxidation of PPy. As a result, bare PPy powder is thoroughly burn off at 600 °C. After TGA test, the bare urchin-like MnO_2_ sample and caddice-clew-like MnO_2_ sample remain 88.7wt.% and 91.6% at 800 °C. The most weight loss is in the temperature range of 60–300 °C, so it can be ascribed to the removal of surface absorbed solvents, although both samples looked very dry. For 30 μL PPy-coated urchin-like MnO_2_ sample, the weight loss in the range of 60–260 °C is 10%, and the whole weight loss in the range of 0–800 °C is 32.3%. The change in weight before and after the oxidation of PPy can be directly translated into the amount of PPy in the MnO_2_@PPy sample [25]. Using this method, the amounts of PPy in the 30 μL PPy-coated urchin-like MnO_2_ sample is about 22%. This value is close to the theoretical amounts of PPy. For 50 μL PPy-coated caddice-clew-like MnO_2_ sample, the whole weight loss in the range of 0–800 °C is 43.9% and the weight loss in the range of 60–260 °C is 14%. So, the actual amounts of PPy in 50 μL PPy-coated caddice-clew-like MnO_2_ sample is about 30% which is much near to the theoretical value. Therefore, the results confirm that the MnO_2_ particles are covered by the PPy organic film.

### XRD Characterization of Samples

**Fig. 5:**
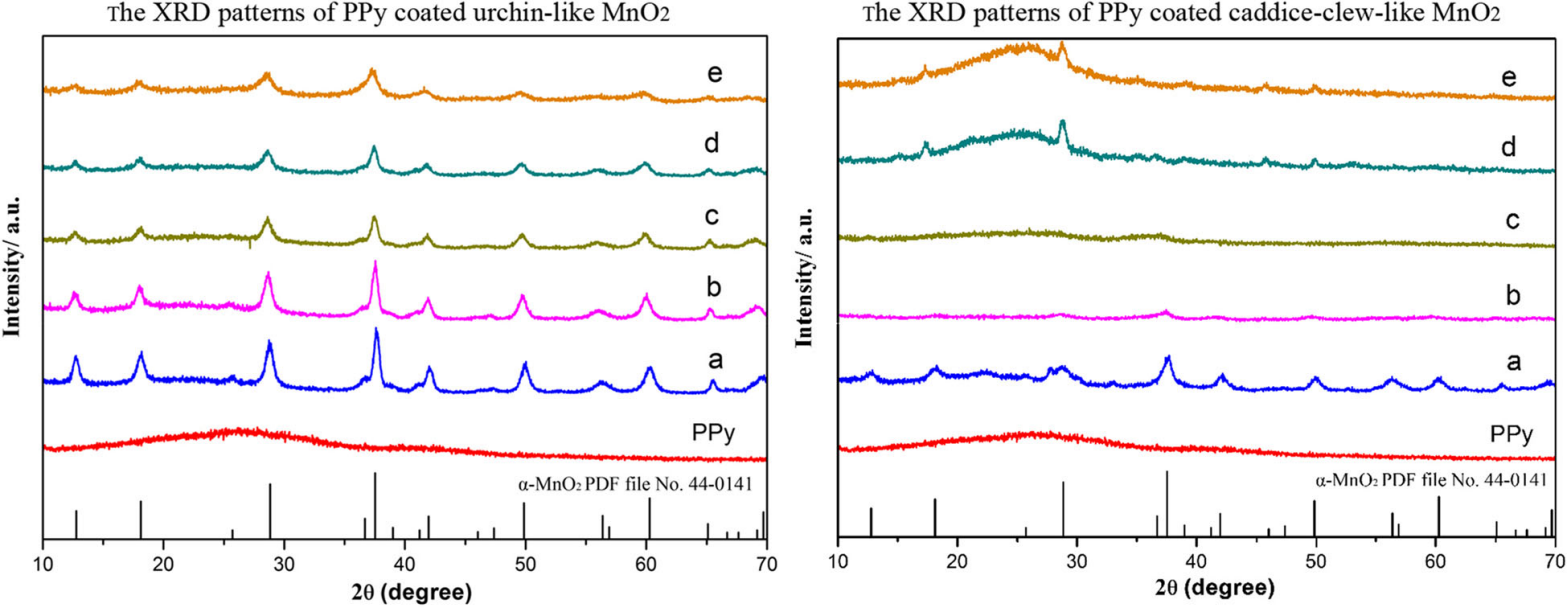
The XRD patterns of PPy-coated MnO_2_ samples. The left is (**a**) urchin-like MnO_2_ sample and (**b**) 10 μL, (**c**) 20 μL, (**d**) 30 μL, and (**e**) 50 μL PPy-coated. The right is (**a**) caddice-clew-like MnO_2_ sample and (**b**) 30 μL, (c) 50 μL, (**d**) 75 μL(**e**), and 100 μL PPy-coated

The crystalline structures of MnO_2_@PPy samples are examined by XRD (Fig. 5). As shown, PPy is an amorphous structure. When coated by PPy, the urchin-like MnO_2_@PPy samples retain the α-MnO_2_ structure. The diffraction peaks appear at 2θ = 12.7°, 18.1°, 28.8°, 37.5°, 42.1°, 49.9°, 56.2°, and 60.3° match well with the diffraction peaks of (110),(200),(310),(211),(301),(411),(600), and (521) crystal planes of α-MnO_2_ standard data (JCPDS card PDF file No. 44-0141). With the increasing of the amount of PPy, the intensity of XRD peaks decrease gradually due to the formation of amorphous PPy. As shown in the PPy-coated caddice-clew-like MnO_2_ samples, there are obvious amorphous peaks from 15° to 30° in 75 and 100uL samples. When coated by PPy, the caddice-clew-like MnO_2_@PPy samples retain α-MnO_2_ structure too. With the increasing of the amount of PPy, the materials obviously change from crystalline to amorphous. These results further prove that the PPy organic film have successfully coated on MnO_2_ particles.

### Electrochemical Performance

**Fig. 6:**
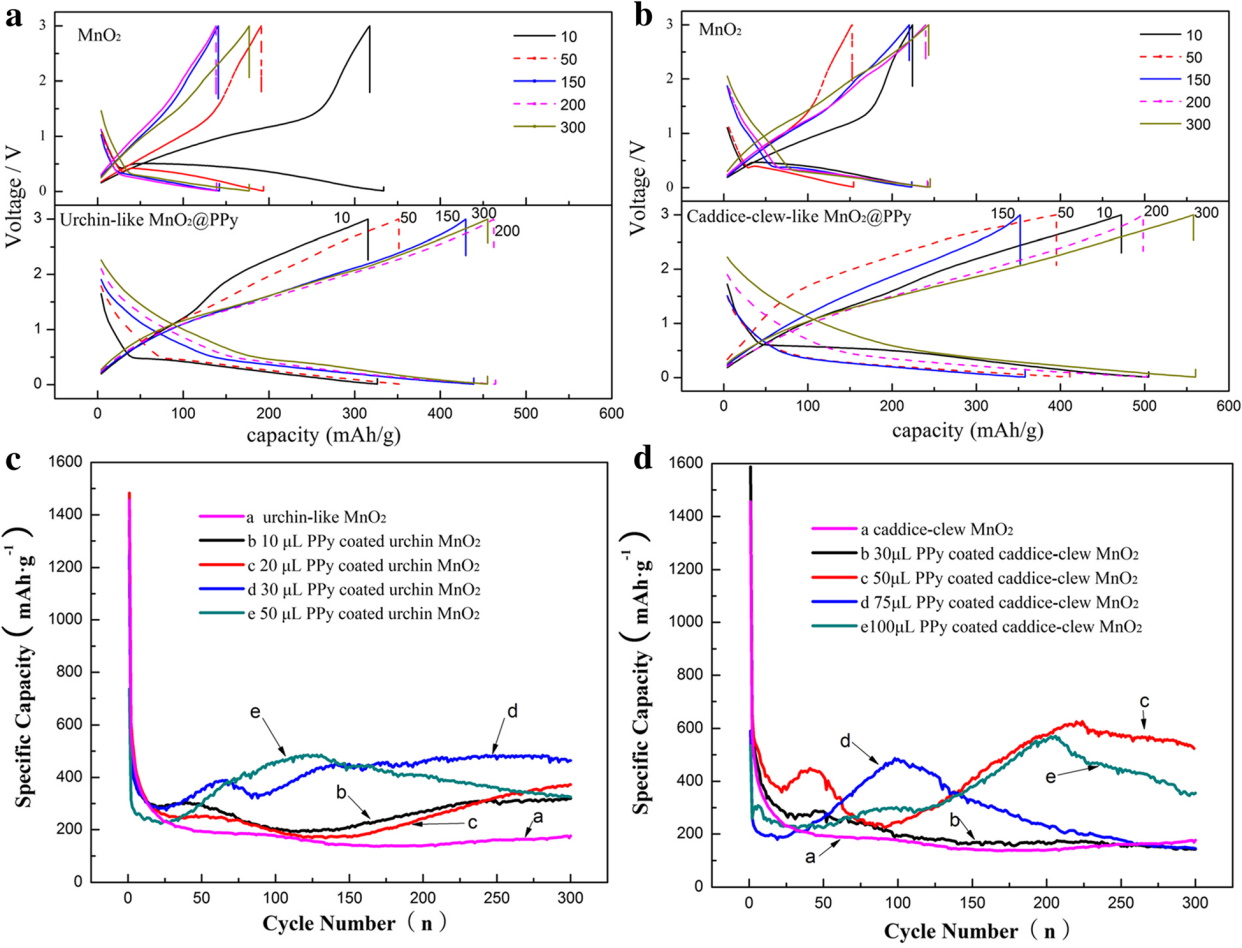
**a**, **b** Charge-discharge curves for selected cycles of 30 μL PPy-coated MnO2 sample and 50 μL PPy-coated caddice-clew-like MnO2 sample. **c**, **d** The cycling performance of the MnO2 sample and PPy-coated MnO2 samples

The electrochemical performances of these MnO_2_@PPy samples as anode materials for LIBs are investigated. Figure 6a, b present the typical charge-discharge curves of the anodes (compared to the full battery) constructed from the bare MnO_2_ sample and MnO_2_@PPy samples at 0.2 C rate in the voltage range of 0.01–3.00 V (vs. Li/Li^+^). For clarity, only the bare MnO_2_ sample and the MnO_2_@PPy with the best charge-discharge performances are shown. As can be seen, the discharge-charge profiles of MnO_2_@PPy samples are similar to those of bare MnO_2_, which indicates that the hybrid products coated by organic PPy shells do not change the electrochemical nature of MnO_2_ LIBs anodes. However, the lithium storage performance of PPy-coated MnO_2_ sample has been improved greatly. The bare urchin-like MnO_2_ sample and PPy-coated urchin-like MnO_2_ sample both have high initial discharge specific capacity as approximate 1200–1400 mAh g^−1^, while the theoretical discharge specific capacity is 1232 mAh g^−1^. The extra discharge specific capacities may result from the formation of SEI layer [14]. After 10 cycles, the discharge specific capacity of bare urchin-like MnO_2_ sample decreases to below 200 mAh g^−1^. As a comparison, the discharge specific capacity of PPy-coated urchin-like MnO_2_ sample remains at about 500 mAh g^−1^ even after 300 cycles. The caddice-clew-like MnO_2_ and the PPy-coated caddice-clew-like MnO_2_ are much similar. After 10 cycles, the discharge specific capacity of bare caddice-clew-like MnO_2_ decreases to below 200 mAh g^−1^. The PPy-coated caddice-clew-like MnO_2_ sample maintains at 500–600 mAh g^−1^ after 300 cycles.

To evaluate their lithium-storage cyclic stability, discharge/charge measurements are performed for 300 cycles on MnO_2_@PPy samples with different pyrroles coated. The thickness of PPy is controlled by the amount of pyrrole. As shown in Fig. 6c, d, when the amount of pyrrole is small (such as 30 uL for caddice-clew-like MnO_2_ and 10 uL for urchin-like MnO_2_), the lithium-storage capacity of this hybrid MnO_2_@PPy sample improves not clearly. This indicates that the PPy film is too thin to prevent MnO_2_ materials suffering from pulverization. However, when the amount of pyrrole increases, the discharge specific capacities of hybrid MnO_2_@PPy samples are remarkably enhanced. For caddice-clew-like MnO_2_, when the amount of pyrrole increases to 50 uL, the hybrid MnO_2_@PPy sample has the biggest discharge specific capacities as 620 mAh g^−1^ after 300 cycles. For urchin-like MnO_2_, the biggest discharge specific capacity appears when 30 uL pyrrole is used. The discharge specific capacity at the 300th cycle is 480 mAh g^−1^. Furthermore, as can be seen from Fig. 6c, d, all the hybrid MnO_2_@PPy samples have improved cyclic stabilities. The improved lithium-storage cyclic stabilities of the hybrid MnO_2_@PPy samples can attribute to the unique structure of the metal oxide/conducting polymer core-shell hybrid products. In this structure, the flexible PPy shell can effectively buffer the structural expansion and contraction of MnO_2_ caused by the repeated embedding and disengagement of the Li ions. In addition, the PPy shell can prevent the pulverization of MnO_2_, as well as protect the loss of electrical contact between the MnO_2_ material and the current collector (copper foil). Whereas, the low capacity and fast capacity fading of bare MnO_2_ can attribute to the pulverization and loss of inter-particle contact of MnO_2_ or the contact of MnO_2_ with copper foil collector due to large volume expansion/contraction during repeated charging-discharging processes. Therefore, this experiment of PPy coating provides an effective way to mitigate the problem of capacity fading of all the transition metal oxide materials as anode materials for LIBs.

**Fig. 7:**
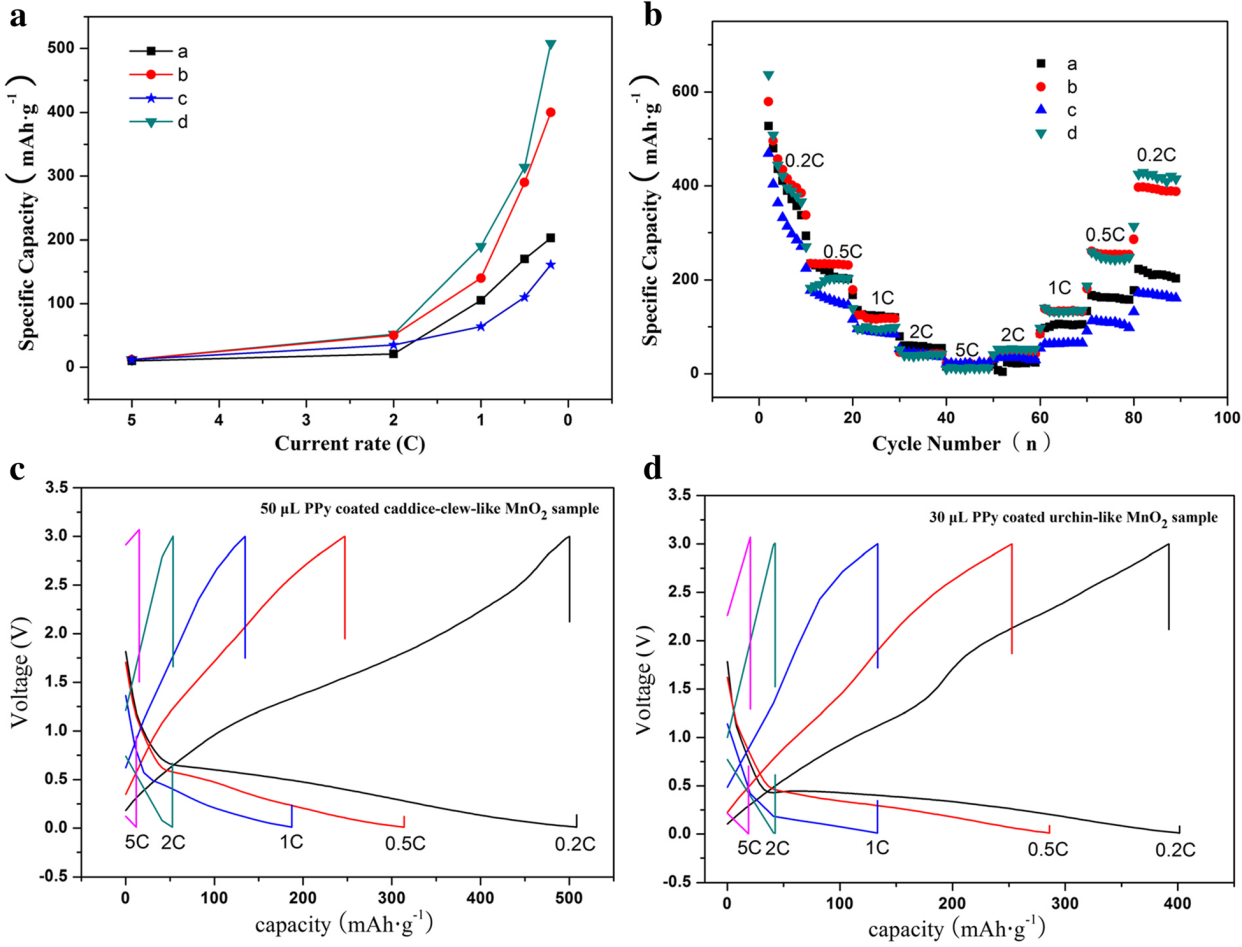
**a** Rate capability, **b** rate performance, and **c**, **d** charge-discharge curves of the MnO_2_@PPy samples. (**a**, **b**) Urchin-like MnO_2_ sample and 30 μL PPy-coated sample. (**c**, **d**) Caddice-clew-like MnO_2_ sample and 50 μL PPy-coated sample

The rate performance of MnO_2_@PPy samples are shown in Fig. 7. To test the rate capability, charge/discharge cycles are performed at the voltage range of 0.01–3.0 V and the discharge rate as 0.2C → 0.5C → 1.0C → 2.0C → 5.0C → 2.0 C → 1.0 C → 0.5C → 0.2C. Figure 7a is the rate capability in the stage from 5.0 to 0.2 C. As shown, the discharge specific capacity of all the MnO_2_ samples in the 5.0 to 0.2 C stage is much similar to that in the stage from 0.2 to 5 C, which proofs that the MnO_2_ samples have relatively high reversibility. However, the discharge specific capacities of all the MnO_2_ samples are poor above the 1 C rate. The merit of the hybrid MnO_2_@PPy samples in the rate performance can be seen at the low rates (0.2, 0.5, and 1 C). After the discharge at 5 C, the discharge capacity of the PPy coated caddice-clew-like MnO_2_ sample is 508 mAh g^−1^ at 0.2 C, while much smaller discharge capacity is obtained as only 160 mAh g^−1^ at 0.2 C of the bare caddice-clew-like MnO_2_ sample. So, the PPy-coated caddice-clew-like MnO_2_ sample has improved rate performance. The situation of the PPy-coated urchin-like MnO_2_ sample is much similar; nevertheless, the discharge capacity is a little lower than that of the PPy-coated caddice-clew-like MnO_2_ sample.

As shown in the rate performance, the urchin-like MnO_2_ micromaterial has relatively higher discharge specific capacity than caddice-clew-like MnO_2_ micromaterial, which is consistent with previous reports [14]. However, after PPy coating, the caddice-clew-like MnO_2_@PPy sample has better lithium-storage cyclic stability. Here, the conjugate degree of the PPy may be one reason. The FT-IR analysis indicates that the PPy conjugate degree of the caddice-clew-like MnO_2_@PPy sample is higher. So, the caddice-clew-like MnO_2_@PPy sample should have better conductivity and better electrochemical performance. To confirm it, the EIS tests are carried out.

**Fig. 8:**
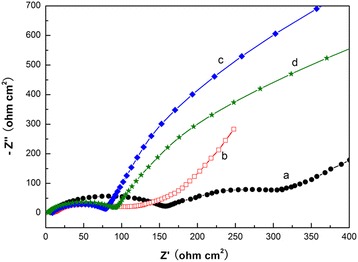
Nyquist plot of Li/MnO_2_ cells at open-circuit voltage. (**a**) caddice-clew-like MnO_2_ sample. (**b**) Urchin-like MnO_2_ sample. (**c**) 50 μL PPy-coated caddice-clew-like MnO_2_ sample. (**d**) 30 μL PPy-coated urchin-like MnO_2_ sample

Figure 8 presents the EIS results for lithium cells after the fifth cycle at an ope-circuit voltage. As shown in Fig. 8a, the impedance spectra of caddice-clew-like MnO_2_ obviously consists of two oblate semicircles in the high-to-medium-frequency region and an inclined line in the low-frequency region. However, the two semicircles of the other three samples are not easily distinguishable. An intercept at the *Z*_real_ axis in the high-frequency region corresponds to the ohmic electrolyte resistance (*R*_s_). The first semicircle in the high frequency ascribes to the Li-ion migration resistance (*R*_sf_) through the SEI films. The second semicircle in the high-to-medium frequency ascribes to the charge transfer resistance (*R*_ct_). The inclined line at low-frequency region represents the Warburg impedance (*W*_s_), which is associated with lithium-ion diffusion in the active material. The semicircular parts of both the hybrid MnO_2_@PPy samples are much smaller than that of the uncoated MnO_2_ sample. This indicates that the conductivities of the hybrid MnO_2_@PPy samples are better and the charge transfer resistance of Li ion decreases after PPy coating. The semicircle resistance of caddice-clew-like MnO_2_@PPy sample is only 77 Ω. The semicircle resistance of urchin-like MnO_2_@PPy sample is only 95 Ω. Here, after PPy coating, the lower resistance of caddice-clew-like MnO_2_ micromaterial can explain the better lithium-storage cyclic stability.

## Conclusions

In summary, MnO_2_@PPy core-shell micromaterials are successfully prepared by chemical polymerization of pyrrole on the MnO_2_ surface. The thickness of the PPy shell can be adjusted by the usage of pyrrole. After formation of MnO_2_@PPy core-shell micromaterials, the cyclic performances as an anode for lithium-ion batteries are improved. Fifty microliters of PPy-coated caddice-clew-like MnO_2_ has the best cyclic performances and has 620 mAh g^−1^ discharge specific capacities after 300 cycles. As a comparison, the discharge specific capacity of bare MnO_2_ materials falls below 200 mAh g^−1^ after 10 cycles. The improved lithium-storage cyclic stability of the MnO_2_@PPy samples can attribute to the core-shell hybrid structure. In this structure, the flexible PPy shell can effectively buffer the structural expansion and contraction of MnO_2_ caused by the repeated embedding and disengagement of Li ions and can prevent the pulverization of MnO_2_. Therefore, this experiment of PPy coating provides us an effective way to mitigate the problem of capacity fading of the transition metal oxide materials as anode materials for LIBs.

## Additional file

Additional file 1: Supporting information. (DOC 3297 kb)
