# Supplementary material for: Preparation of PPy-Coated MnO2 Hybrid Micromaterials and Their Improved Cyclic Performance as Anode for Lithium-Ion Batteries
Source: Nanoscale Res Lett. 2017 Sep 2;12:518. doi: 10.1186/s11671-017-2286-3 (PMC5581745; doi:10.1186/s11671-017-2286-3)

**Supporting Information for**

**Preparation of PPy coated MnO_2_ hybrid micromaterials and their improved cyclic performance as anode for lithium-ion batteries**

Lili Feng^*^, Yinyin Zhang, Rui Wang, Wei Bai, Yanli Zhang, Siping Ji, Zhewen Xuan, Jianhua Yang, Ziguang Zheng, Hongjin Guan

School of Chemistry and Environment, Yunnan Minzu University, Kunming 650500, China; Key Laboratory of Resource Clean Conversion in Ethnic Regions, Education Department of Yunnan, Yunnan Minzu University, Kunming 650500, China

*Corresponding authors: Lili Feng (Email: [lilylian2003@163.com](mailto:lilylian2003@163.com))

**Supporting Information 1**

SEM images of polypyrrole coated caddice-clew-like MnO_2_ sample. In the top left hand corner is pure PPy, (a) caddice-clew-like MnO_2_ sample, (b) 30μL pyrrole coated caddice-clew-like MnO_2_ sample, (c) 50μL pyrrole coated caddice-clew-like MnO_2_ sample, (d) 75μL pyrrole coated caddice-clew-like MnO_2_ sample, (e) 100μL pyrrole coated caddice-clew-like MnO_2_ sample. The scale bar is 1 μm.


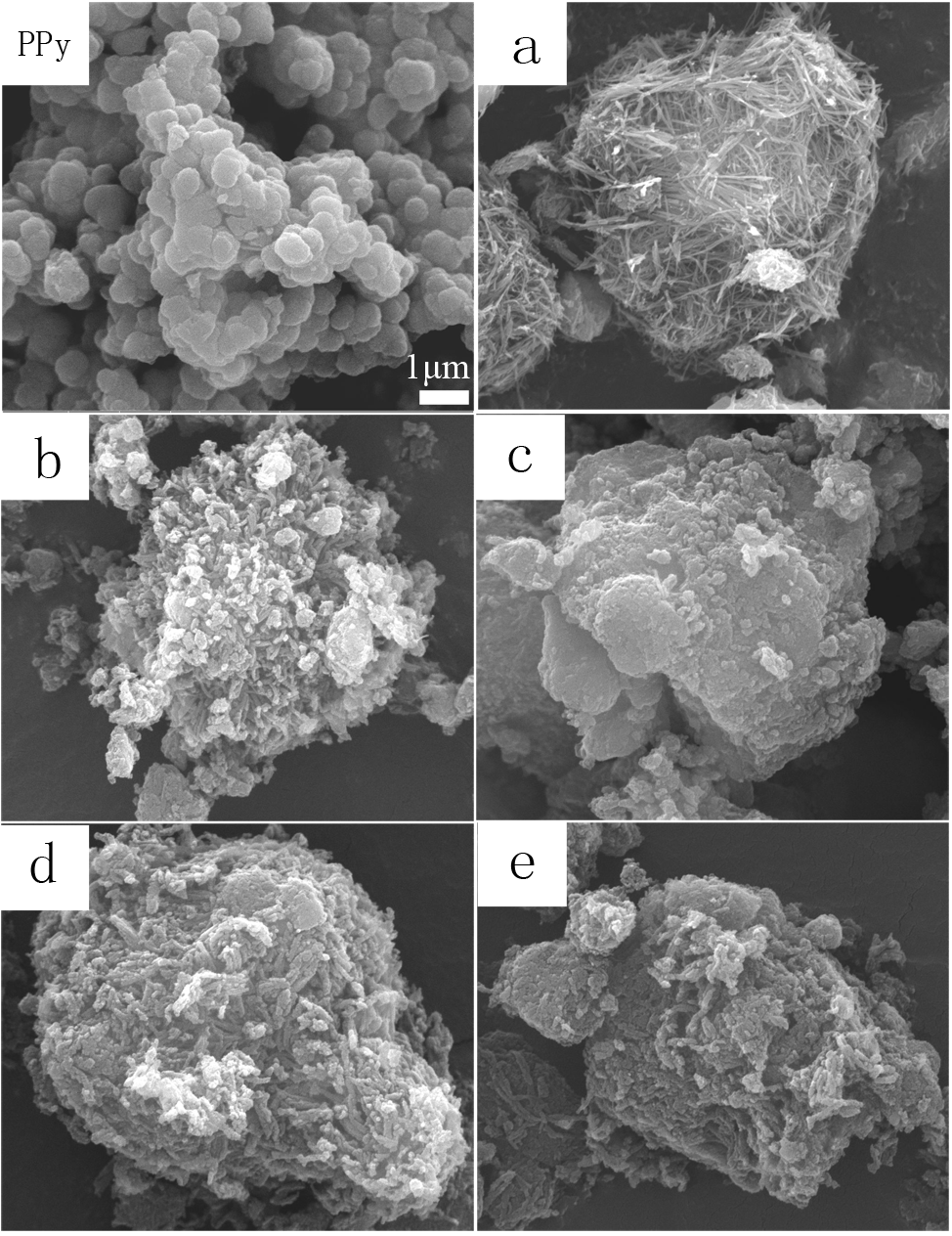


**Supporting Information 2**

SEM images of low magnification of polypyrrole coated caddice-clew-like MnO_2_ samples.


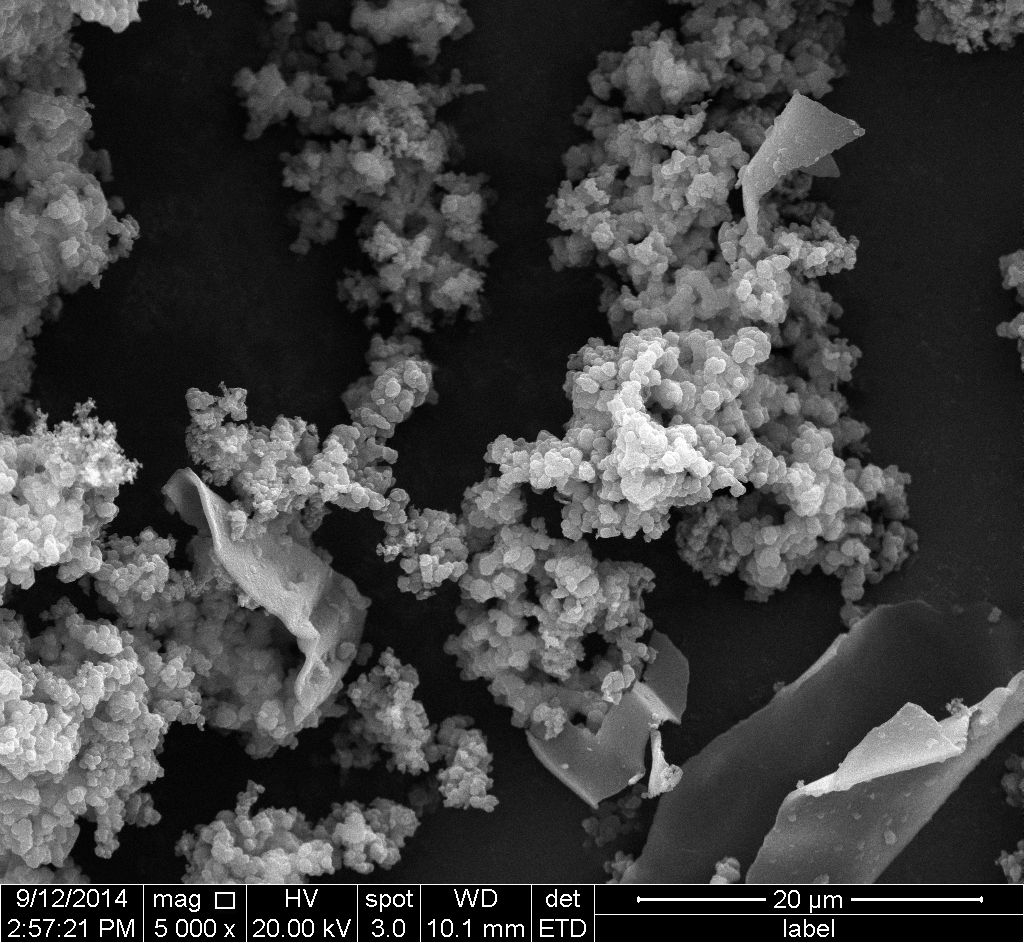

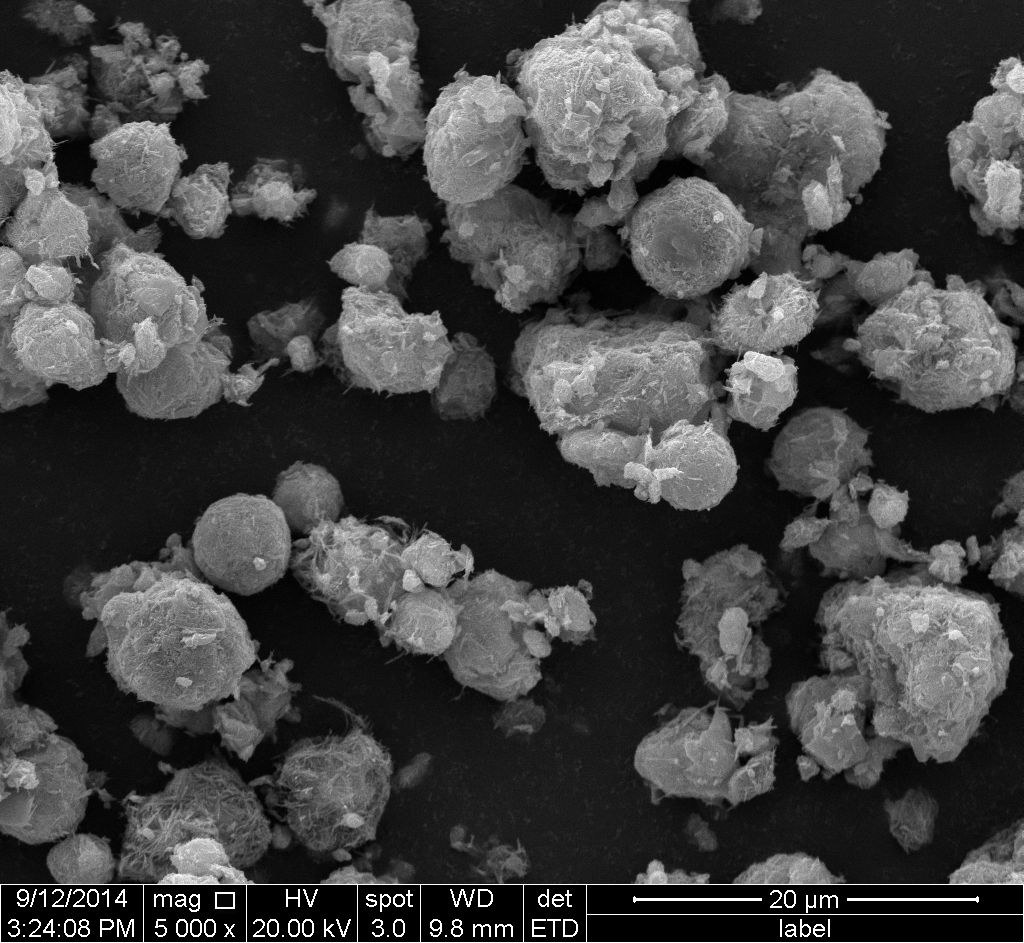


pure PPy caddice-clew-like MnO_2_ sample


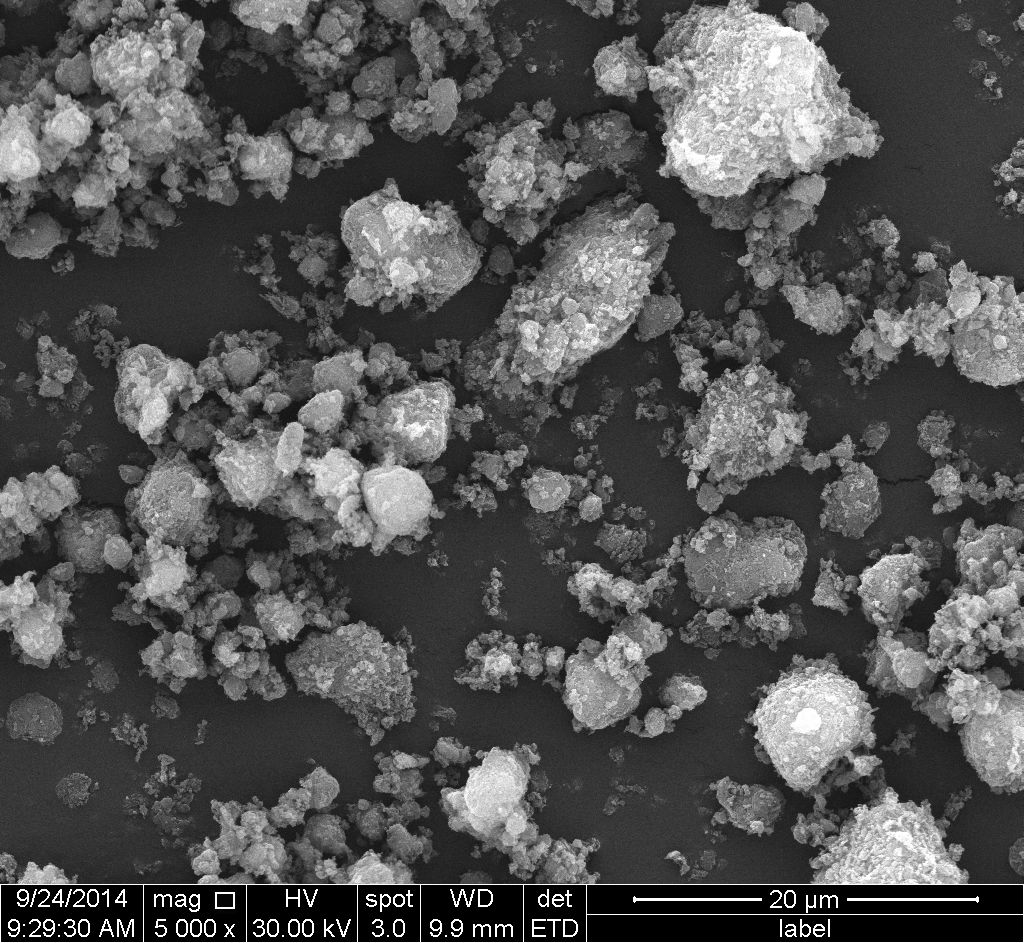

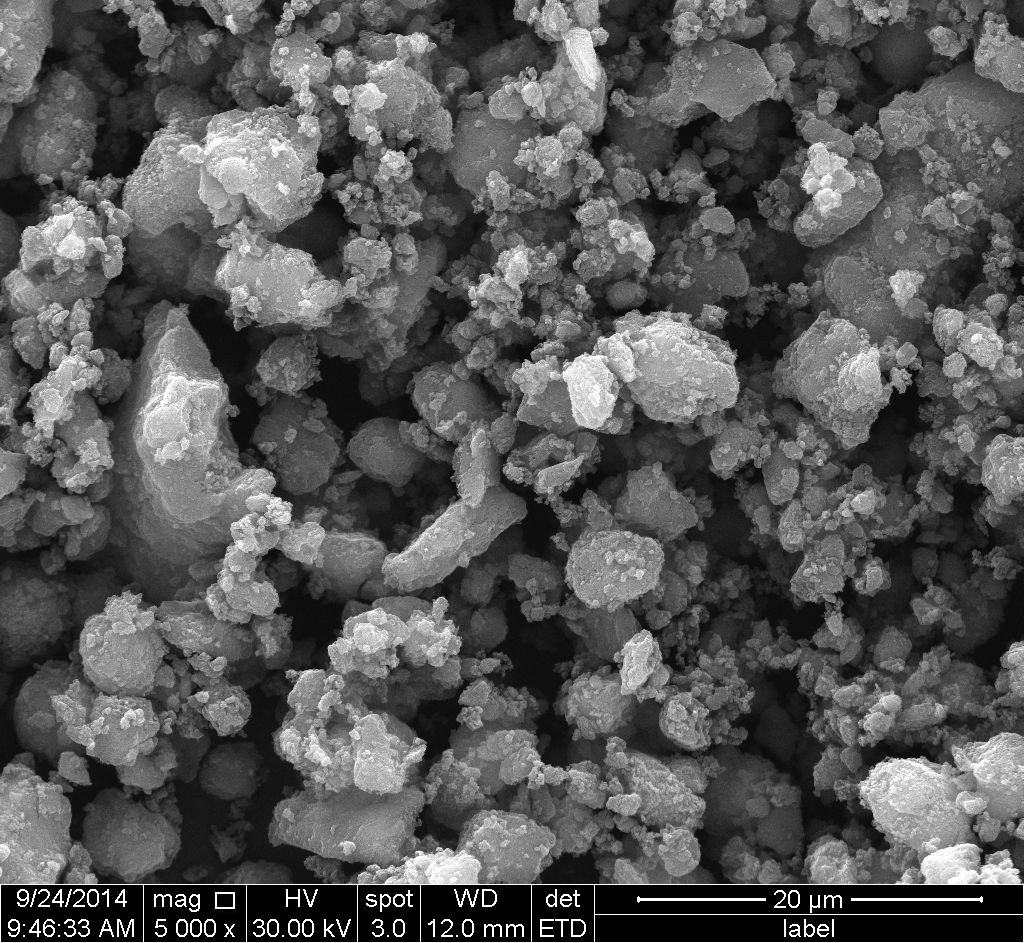


30μL pyrrole coated 50μL pyrrole coated caddice MnO_2_ sample


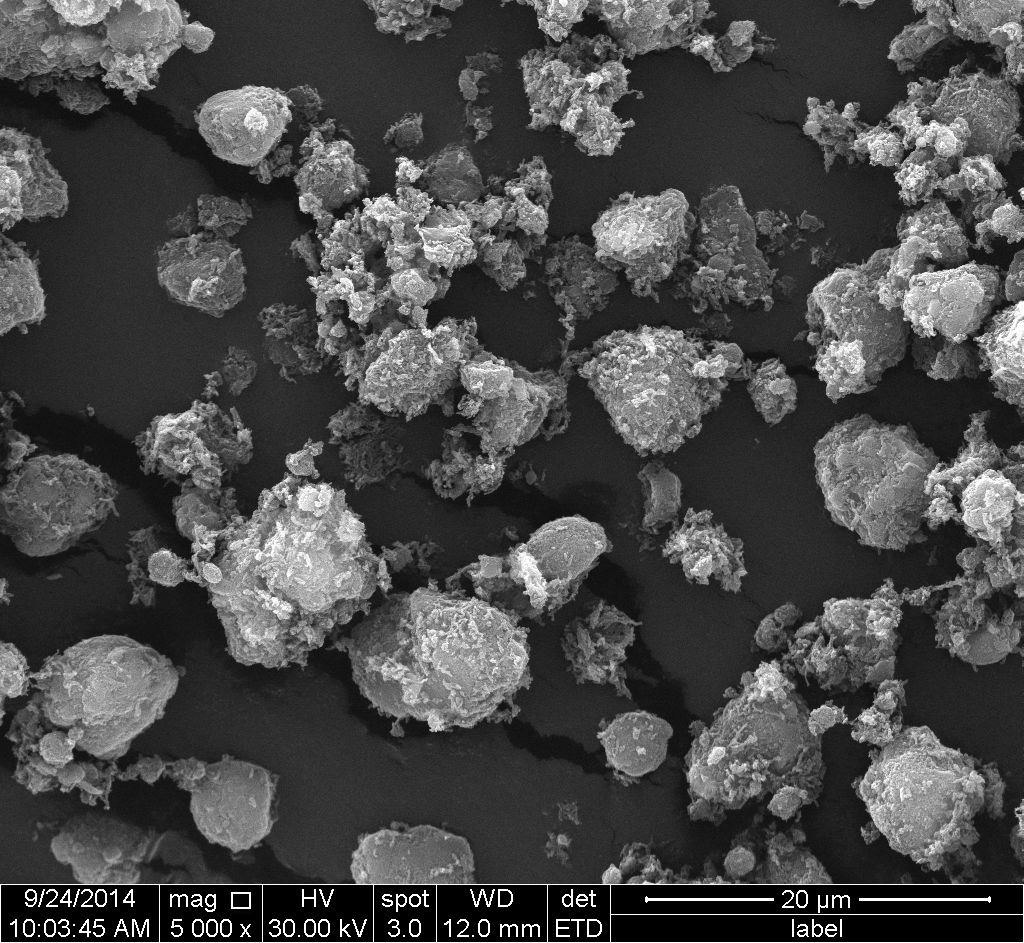

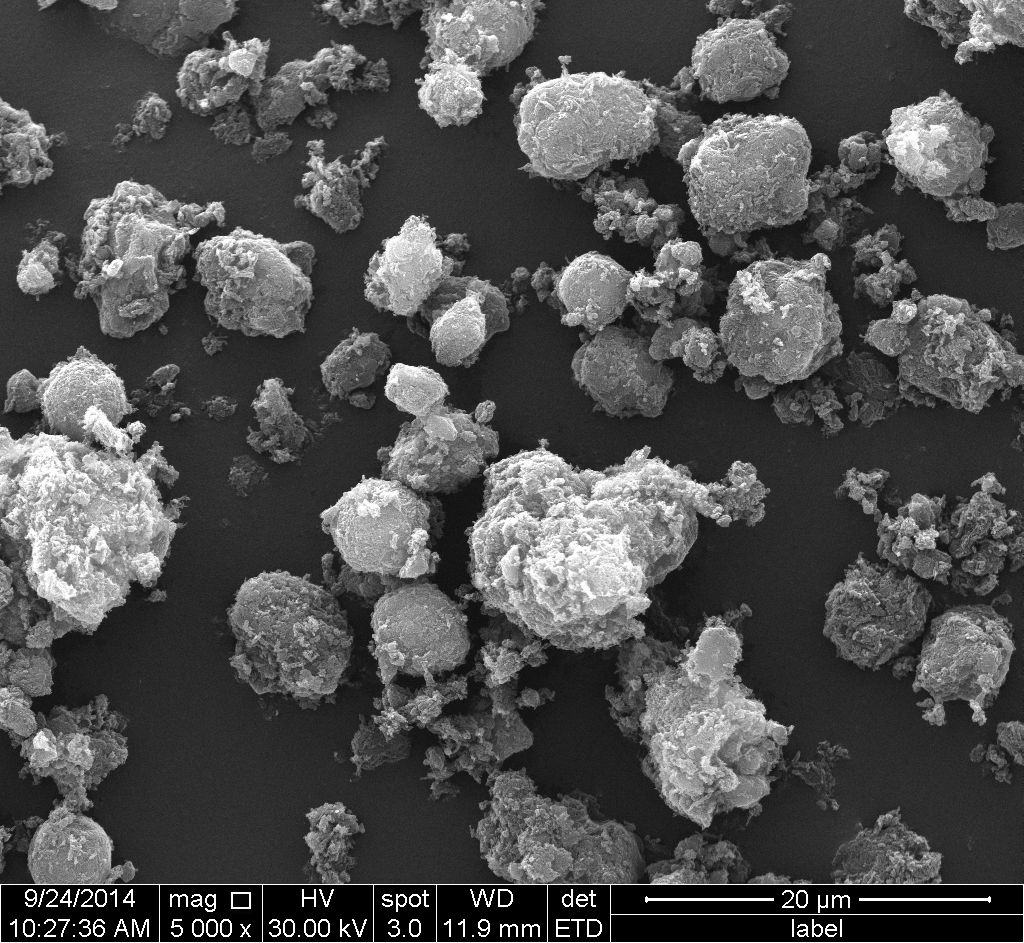


75μL pyrrole coated 100μL pyrrole coated caddice MnO_2_ sample

**Supporting Information 3**

SEM images of low magnification of polypyrrole coated urchin-like MnO_2_ samples.


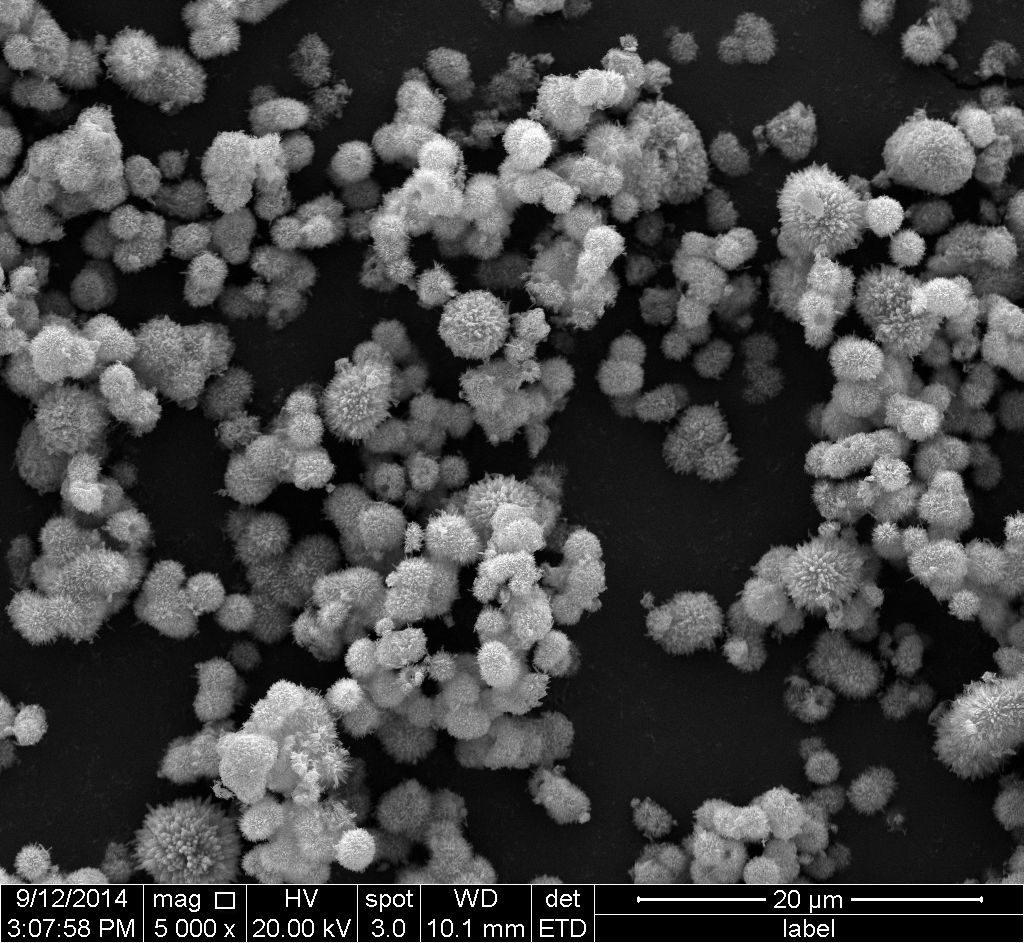

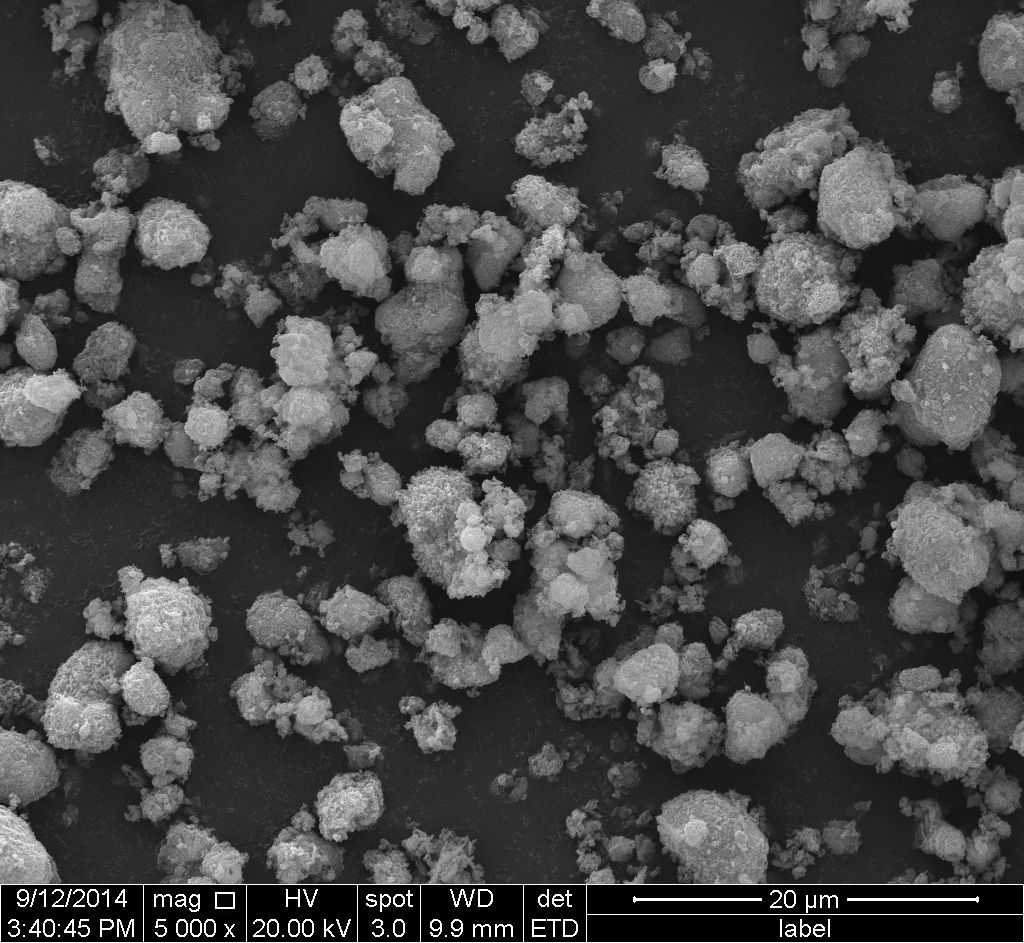


urchin-like MnO_2_ sample 10μL pyrrole coated urchin-like MnO_2_ sample


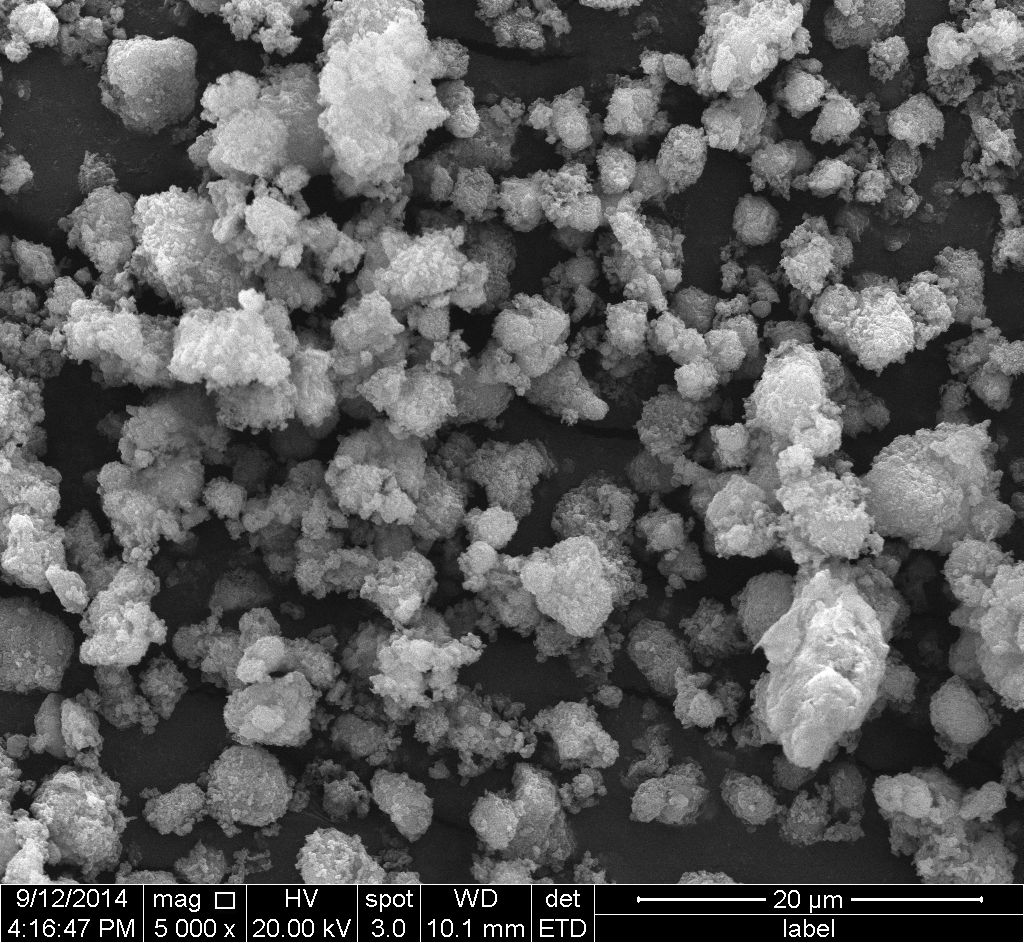

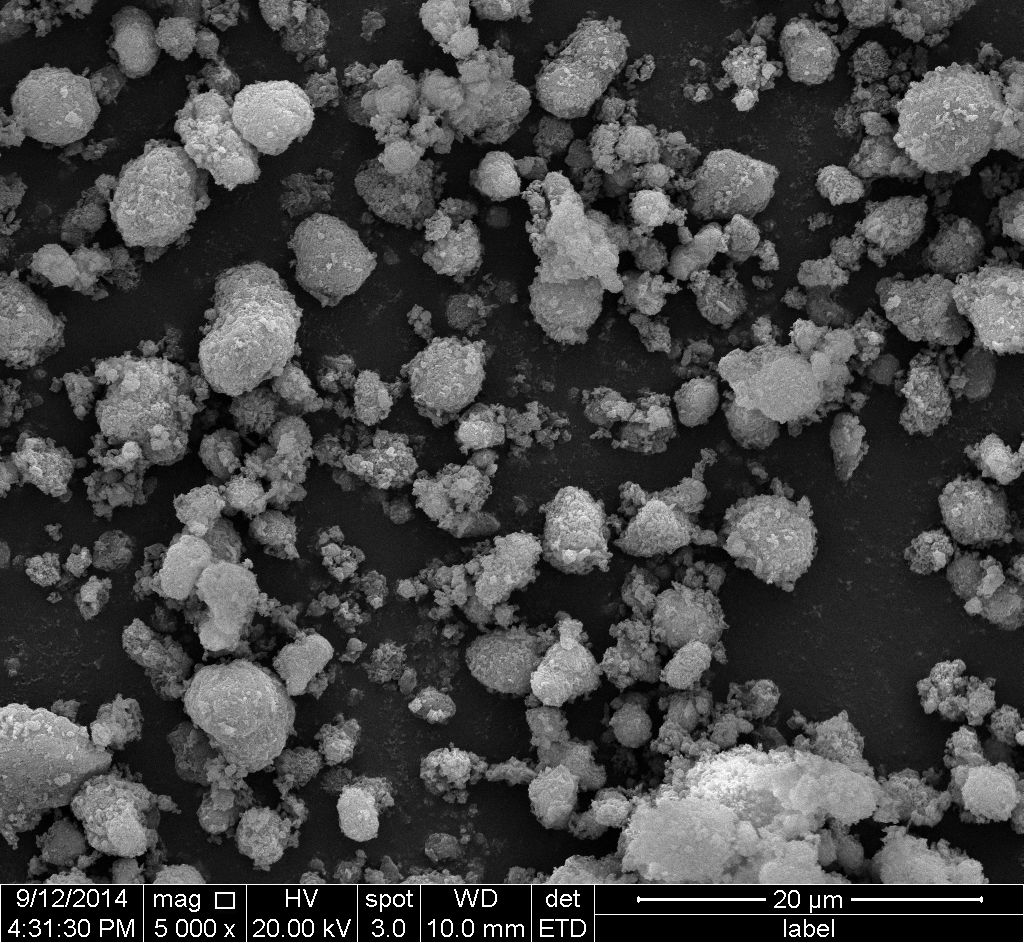


30μL pyrrole coated urchin-like MnO_2_ sample 50μL pyrrole coated urchin-like MnO_2_ sample

**Supporting Information 4**

EDX data and XPS data for caddice-clew-like MnO_2_@PPy sample

| **Element** | **MnO_2_@PPy(30μL) At%** | | **MnO_2_@PPy(50μL)At%** | | **MnO_2_@PPy(100μL)At%** | |
| --- | --- | --- | --- | --- | --- | --- |
|  | EDX | XPS | EDX | XPS | EDX | XPS |
| **C** | **17.46** | **33.8** | **34.72** | **51.2** | **60.96** | **56.7** |
| **N** | **7.84** | **2.7** | **8.83** | **9.6** | **14.63** | **10.8** |
| **O** | **31.38** | **61.5** | **17.46** | **34.3** | **14.28** | **26.7** |
| **Mn** | **35.91** | **2.0** | **27.56** | **2.4** | **3.32** | **5.4** |

**Supporting Information 5**

XPS spectra of 10 μL PPy coated urchin-like MnO_2_ sample


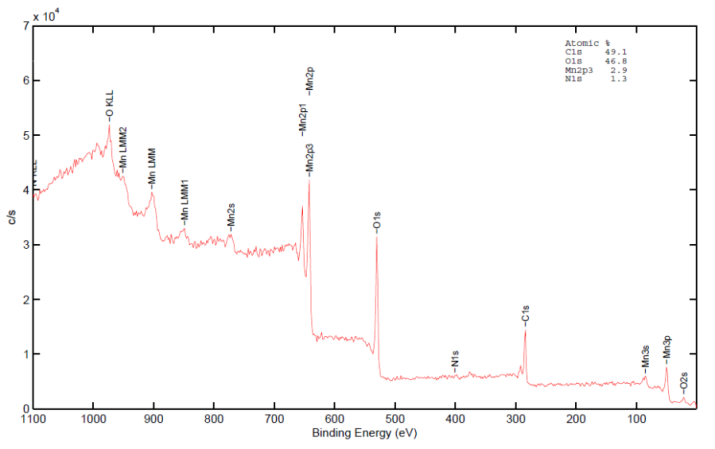


XPS spectra of 30 μL PPy coated urchin-like MnO_2_ sample


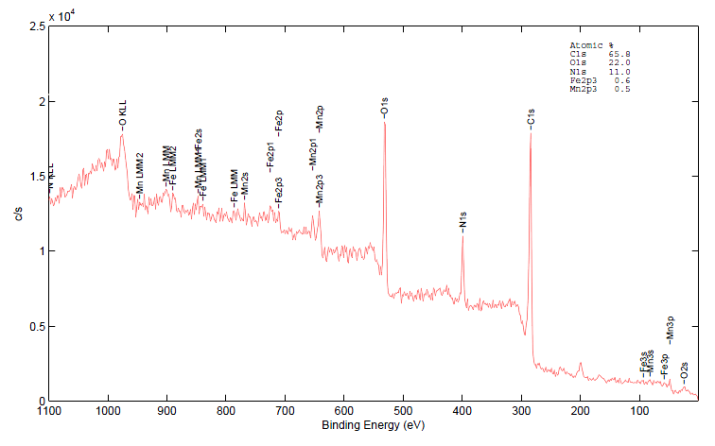


XPS spectra of 50 μL PPy coated urchin-like MnO_2_ sample


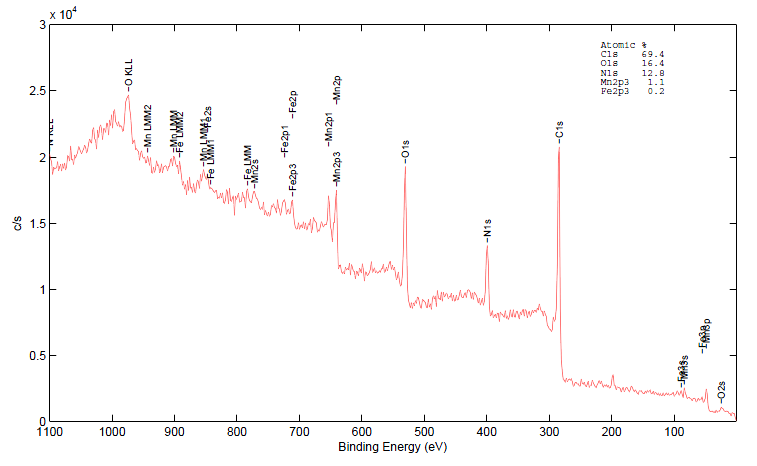


XPS spectra of 30 μL PPy coated caddice-clew-like MnO_2_ sample.


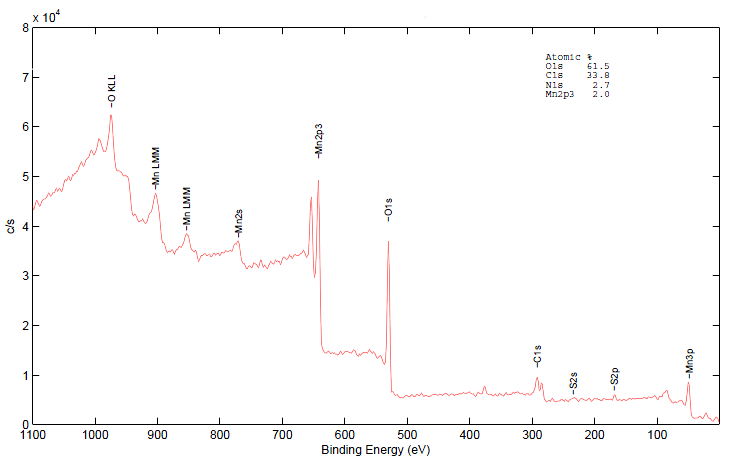


XPS spectra of 50 μL PPy coated caddice-clew-like MnO_2_ sample.


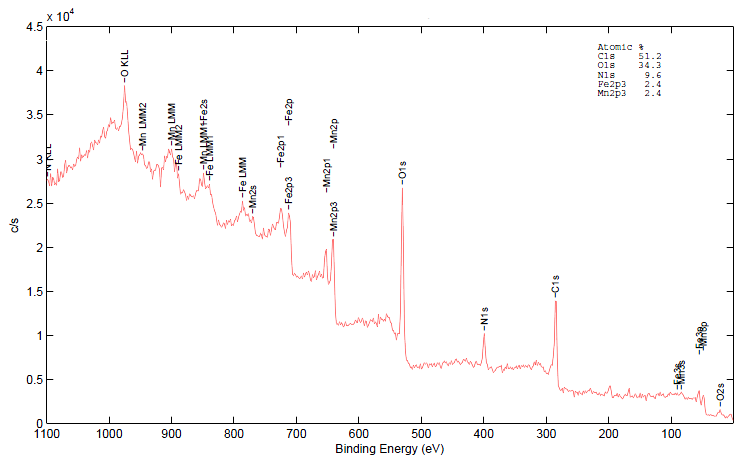


XPS spectra of 100 μL PPy coated caddice-clew-like MnO_2_ sample.


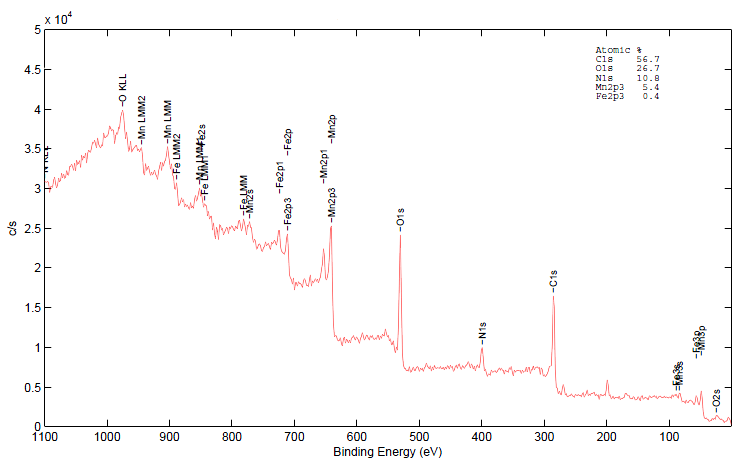

Supplement: Additional file 1: — Supporting information. (DOC 3297 kb) [file 11671_2017_2286_MOESM1_ESM.doc]
